# ARID1A-Deficient and 11q13-Amplified Metastatic Pancreatic Cancer Initially Presenting as Retroperitoneal Fibrosis in a Patient with Familial CHEK2 Variant

**DOI:** 10.3390/diagnostics15161998

**Published:** 2025-08-09

**Authors:** Siming Kong, Tongxin Cao, Yakun Liu, Xuedong Wang, Mingshuo Wang, Tianzi Luo, Yunfang Wang, Pengfei Wang, Hui Bai

**Affiliations:** 1 Center for Clinical and Translational Science, Beijing Tsinghua Changgung Hospital, School of Clinical Medicine, Tsinghua Medicine, Tsinghua University, Beijing 102218, China; ksm09410@163.com (S.K.); ctxa05070@btch.edu.cn (T.C.); lyka04896@btch.edu.cn (Y.L.); wmsa04626@btch.edu.cn (M.W.); ltza04897@btch.edu.cn (T.L.); wyfa02717@btch.edu.cn (Y.W.); 2 Hepato-Pancreato-Biliary Center, Beijing Tsinghua Changgung Hospital, School of Clinical Medicine, Tsinghua Medicine, Tsinghua University, Beijing 102218, China; wxda01026@btch.edu.cn

**Keywords:** retroperitoneal fibrosis, secondary retroperitoneal fibrosis, malignancy-associated retroperitoneal fibrosis, cancer of unknown primary, molecular profiling

## Abstract

**Background and Clinical Significance**: Retroperitoneal fibrosis (RPF), a rare fibroinflammatory disorder, is classified into idiopathic (iRPF) and secondary (sRPF) forms, with the latter posing significant diagnostic challenges in routine clinical pathway due to atypical presentations, especially in malignancy-associated (maRPF) cases. **Case Presentation**: Here, we report a 38-year-old female with congenital pancreatic hypoplasia presenting with elusive hypometabolic retroperitoneal masses, initially suggestive of iRPF. Persistent CA19-9 elevation prompted histopathological evaluation, revealing poorly differentiated adenocarcinoma of indeterminate origin. Timely integrated molecular profiling identified maRPF secondary to metastatic pancreatic adenocarcinoma, revealing rare genomic alterations, including a truncating *ARID1A* mutation NM_006015:c.4336C>T (p. R1446*) and *CCND1/FGF3/FGF4/FGF19* (11q13) co-amplification, which resolved diagnostic ambiguity and delineated disease biology. Despite identifying these molecular features, poor prognosis was predicted, and no clinically actionable targets were detected, underscoring the need for future therapeutic development. **Conclusions**: This paradigm highlights molecular profiling as a critical adjunct to conventional diagnostics in maRPF, bridging the gap between histopathological ambiguity and biologically grounded clinical decision-making.

## 1. Introduction

Retroperitoneal fibrosis (RPF) is a rare disorder with an annual incidence of 0.1–1.3 per 100,000 people [1]. The key features of RPF include chronic inflammation, excess proliferation of fibroblasts, and extracellular matrix deposition in the retroperitoneal area [2]. RPF can be classified as idiopathic retroperitoneal fibrosis (iRPF) and secondary retroperitoneal fibrosis (sRPF) [3]. iRPF accounts for approximately 70% of cases and is most commonly found in individuals aged 40–60 years [4,5]. Patients often present with dull lower back pain, sometimes radiating to the abdomen or groin, which is often alleviated by aspirin. Other symptoms include weight loss, anorexia, nausea, malaise, fever, hypertension, and oliguria or anuria. iRPF is linked to IgG4-related disease in 35–60% of cases and may present alone or in association with other autoimmune conditions [4]. The typical iRPF mass, centered around the abdominal aorta at the L4 to L5 level, encircles the ureters, causing hydronephrosis due to disrupted peristalsis [5]. Compression of the inferior vena cava can lead to thrombosis and edema [6]. Proximal extension may cause renal vein hypertension and hematuria. Glucocorticoid therapy remains the first-line treatment, with an overall 80% favorable clinical response rate [7].

sRPF accounts for approximately 30% of RPF, and malignancy-associated RPF (maRPF) is even rarer, for about 8% of sRPF [7]. maRPF is most commonly associated with tumors such as carcinoid, lymphoma, sarcoma, prostate, and gastric cancers [8]. sRPF to pancreaticobiliary malignancies is exceedingly rare and may follow a distinct pathogenic pathway beyond direct tumor invasion [9,10]. Proposed mechanisms include tumor-induced desmoplastic responses, chronic inflammatory signaling, and paracrine cytokine activity, which promote excessive fibroblast activation and diffuse fibrosis rather than the formation of discrete tumor masses [11]. The exact etiology and pathological mechanisms of maRPF remain poorly understood, therefore complicating early diagnosis and often leading to misinterpretation as benign iRPF [12]. Imaging methods such as computed tomography (CT) and magnetic resonance imaging (MRI) play a critical role in detecting retroperitoneal fibrotic plaques and assessing their impact on surrounding vascular and urinary structures [12,13]. However, in some cases, these techniques lack specificity in differentiating benign from malignant causes [14]. Positron emission tomography–computed tomography with ^18^F-fluorodeoxyglucose (^18^F-FDG PET-CT) is often used to evaluate metabolic activity, but its diagnostic utility is limited in fibrotic tumors due to variable uptake patterns [15].

## 2. Case Presentation

### 2.1. Clinical Findings

A 38-year-old woman presented to our department with a two-month history of fatigue, anorexia, and postprandial abdominal distension in December 2022. Two years earlier, she had undergone a laparoscopic cholecystectomy with choledocholithotomy, during which heterotaxy polysplenia syndrome was diagnosed. The patient had recently recovered from COVID-19 and reported the use of traditional Chinese medicine but denied any other recent medication use. Her family history was notable for situs inversus in her sister and breast cancer in her mother. On admission, significant laboratory abnormalities were observed. Liver function tests revealed significant elevations in alanine aminotransferase (ALT, 195.8 U/L; normal, 7–40 U/L), aspartate aminotransferase (AST, 148.6 U/L; normal, 13–25 U/L), total bilirubin (34.9 μmol/L; normal, 0–21 μmol/L), and direct bilirubin (30.5 μmol/L; normal, ≤8 μmol/L). Elevated levels of CA 19-9 (1200 U/L to 1334.32 U/L; normal, <37 U/L) and protein induced by vitamin K absence or antagonist-II (PIVKA-II, 1045.22 mAU/mL; normal, <40 mAU/mL) were also observed. Moreover, erythrocyte sedimentation rate (ESR, 77 mm/h; normal for females, 0–20 mm/h), C-reactive protein (CRP, 29.43 mg/L; normal, <5 mg/L), and positive anti-nuclear antibodies were markedly altered. Initially, a diagnosis of heterotaxy polysplenia syndrome with iRPF was considered. However, the progression of obstructive jaundice, persistent weight loss, and an increase in CA 19-9 levels raised suspicion for malignancy. Subsequent repeated biopsies confirmed retroperitoneal fibrosis secondary to adenocarcinoma, with a suggested primary origin in the pancreaticobiliary system.

### 2.2. Radiologic Assessment

**Figure 1:**
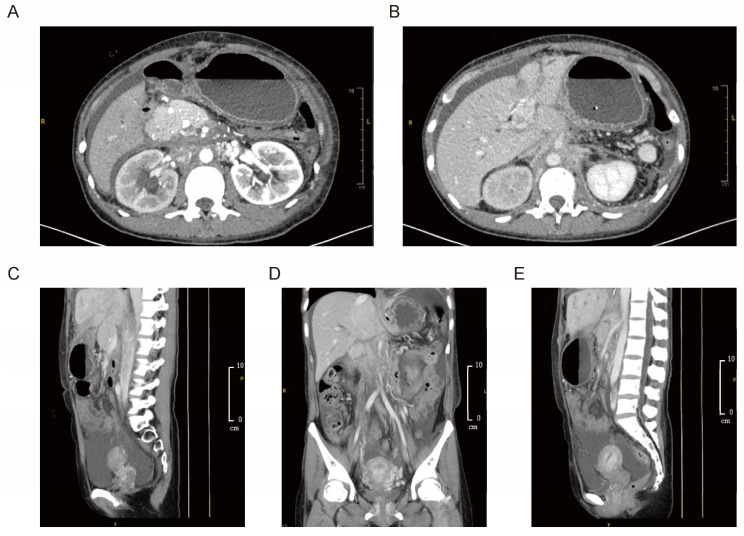
Imaging findings of retroperitoneal fibrosis with peripancreatic and hepatic involvement of the patient. (**A**,**B**) Abdominal contrast-enhanced CT revealed duodenal compression by a congenitally enlarged pancreatic head, stricture of the hepatic portal bile duct, and dilation of second- and third-order bile ducts on the right. The bile duct wall appeared thickened with contrast enhancement. (**C**–**E**) Coronal and sagittal contrast-enhanced CT demonstrated thickening and enhancement of the bile duct wall. Space-occupying lesions in the retroperitoneal and peripancreatic regions extended toward the round ligament of the liver, exhibiting a fibrosis-like appearance rather than a well-defined mass.

CT revealed absence of the inferior vena cava, polysplenia, intrahepatic ductal dilatation, thickening of the gastric fundus, and a mass in the retroperitoneal region. Subsequent magnetic resonance cholangiopancreatography (MRCP) and computed tomography angiography (CTA) showed thrombosis of the left portal vein, congenital pancreatic hypoplasia, and peripancreatic space-occupying lesions. Compression of the duodenum by a congenitally enlarged pancreatic head, stricture of the hepatic portal bile duct, dilation of the level-two and level-three bile ducts on the right side, and thickening with enhancement of the bile duct wall were also confirmed. Contrast-enhanced CT and repeated MRCP upon referral revealed duodenal compression by an enlarged pancreatic head, hepatic bile duct strictures, and thickened, enhancing bile duct walls suggestive of obstruction (Figure 1). PET-CT demonstrated only mildly elevated metabolic activity in lesions around the ligamentum teres hepatis, which was not sufficient to establish a definitive diagnosis of malignancy.

### 2.3. Operative and Histopathologic Evaluation

**Figure 2:**
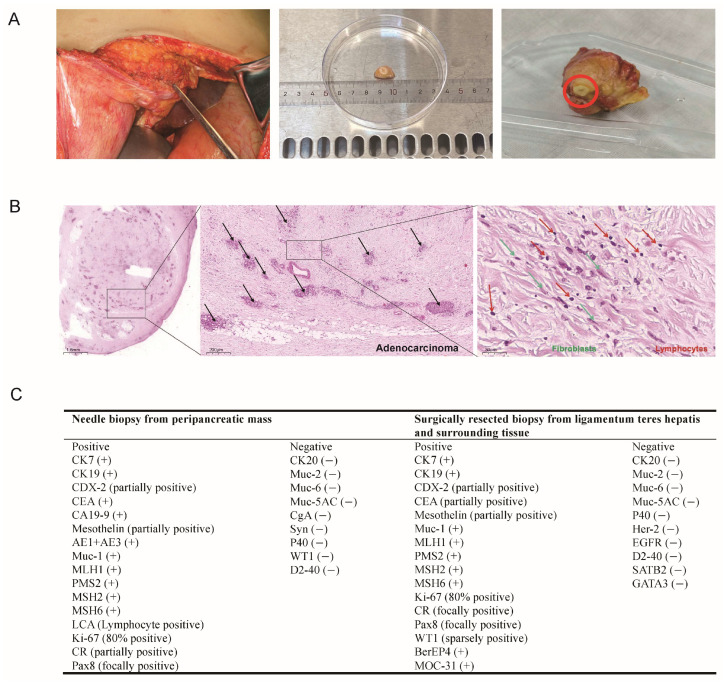
Intraoperative and pathological findings of the patient. (**A**) The intraoperative resected lesion. A hard, immobile ligamentum teres hepatis and surrounding tissue (measuring approximately 6 × 5 × 3 cm, grayish-yellow) were completely excised for histopathological evaluation. (**B**) Histopathological examination of the excised tissue showed scattered tumor cell clusters. The black arrows highlight invasive adenocarcinoma glands scattered within fibrotic stroma, indicating malignancy and tissue invasion. The tumor exhibits poorly differentiated adenocarcinoma with glandular, cord-like, and nested growth patterns. Tumor cells show moderate atypia, accompanied by fibroblast proliferation (green arrows) and lymphocytic infiltration (red arrows), indicative of an inflammatory tumor microenvironment. (**C**) Comprehensive immunohistochemistry (IHC) evaluation confirmed the diagnosis of RPF secondary to adenocarcinoma with primary origination from pancreaticobiliary system.

Ultrasound-guided needle biopsy was first performed on the peripancreatic mass and pathology report revealed poorly differentiated adenocarcinoma, with suggested primary origination from pancreaticobiliary system (Figure 2A). The patient underwent laparoscopy with conversion to open surgery for gastrojejunostomy. Intraoperative findings included ascites, intra-abdominal adhesions, cholestasis in the liver, and varicose omentum and mesenteric veins prone to bleeding. Extensive fibrosis at the hepatic hilum and peripancreatic tissues precluded bile duct dissection and curative surgical removal. Therefore, hard immobile ligamentum teres hepatis and surrounding tissue (6 × 5 × 3 cm, grayish-yellow) were completely excised for histopathological evaluation. Tumor cells within the fibro-adipose tissue demonstrated cord-like, glandular, and nested growth patterns, with moderate cellular atypia and lymphocytes infiltration (Figure 2B). Immunohistochemical staining (IHC) again confirmed the suggested tumor origin as pancreaticobiliary system (Figure 2C).

### 2.4. Tumor Origin Detection, Gene Mutation Identification, and Prognosis Assessment

**Figure 3:**
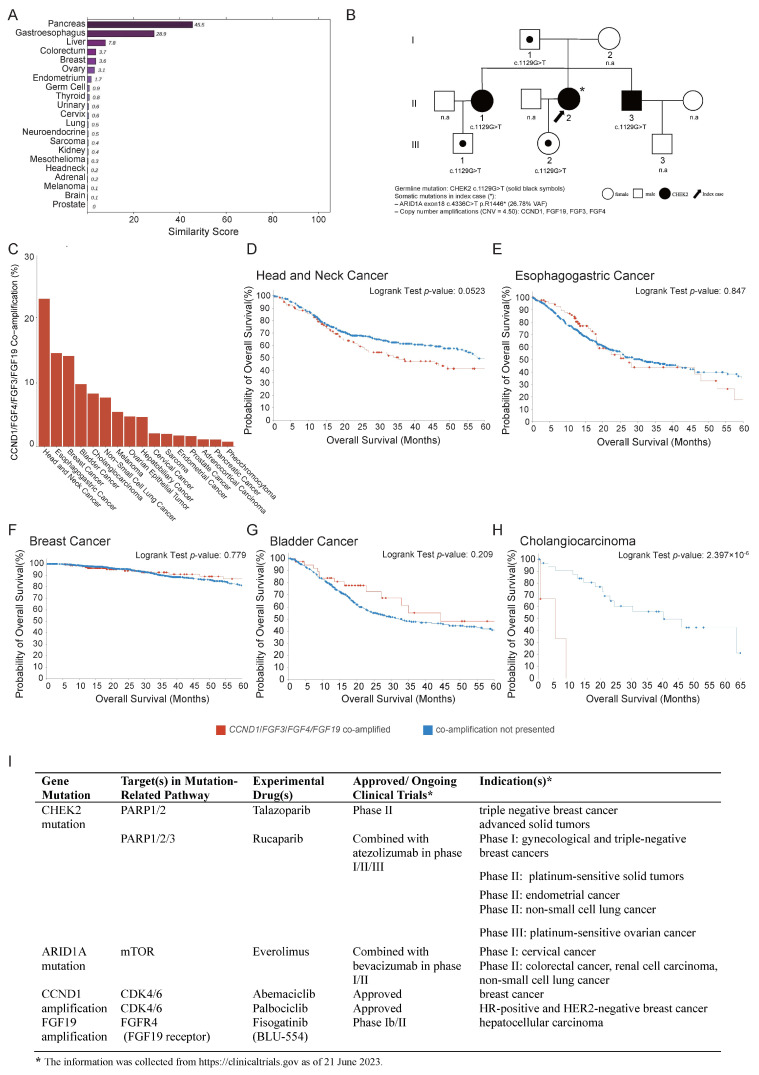
Tumor tissue origin identification. (**A**) A 90-gene panel test (Canhelp-Origin) assay results indicated that tumor on ligamentum teres hepatis was metastatic pancreatic cancer with a similarity score of 45.5. (**B**) Pedigrees of proband’s family indicating the affected individuals (filled in black) inherited a germline *CHEK2* variant and somatic mutations including *ARID1A* (NM_006015: c. 4336C>T (p. R1446*)) and amplifications of *CCND1*/*FGF3*/*FGF4*/*FGF19*. “*” indicates the proband; “n.a.” denotes unaffected (healthy) family members. Roman numerals I, II, and III represent the first, second, and third generations of the family, respectively. (**C**) Genomic alteration frequency of *CCND1*/*FGF3***/***FGF4*/*FGF19* across cancer types from TCGA PanCancer Atlas. (**D**–**H**) Overall survival (OS) across the top 5 frequent cancer types. Potential targeted therapies associated with *CHEK2* germline mutation, *ARID1A* mutation, and *CCND1*/*FGF3*/*FGF4*/*FGF19* co-amplification. (**I**) Experimental approved drugs include Talazoparib and Rucaparib (PARP inhibitors), Everolimus (mTOR inhibitor), Abemaciclib and Palbociclib (CDK4/6 inhibitors), and Fisogatinib (FGFR4 inhibitor). While these therapies are not yet standard of care for pancreatic adenocarcinoma, they are under active investigation across multiple tumor types, highlighting the potential for precision oncology approaches in genomically defined cases. The information was collected from https://clinicaltrials.gov as of 21 June 2023.

To further investigate the tumor origin and refine the differential diagnosis, molecular profiling was performed using a 90-gene expression classifier (Canhelp-Origin), which indicated pancreatic adenocarcinoma as the most likely cancer type with a similarity score of 45.5 (Figure 3A). Targeted sequencing via the ChosenPace panel identified a *CHEK2* germline variant (NM_007194: c. 1129G>T (p. E377*)) in both the index patient and her sister, inherited from their father (Figure 3B). Additionally, analysis of the tumor tissue identified five class II somatic variants, including an *ARID1A* mutation (NM_006015: c. 4336C>T (p. R1446*)) with an estimated copy number of 26.78, and amplifications of *CCND1*/*FGF3*/*FGF4*/*FGF19*, each with a copy number of 4.50. The tumor mutation burden (TMB) was measured at 6.58 mutations/Mb, corresponding to the 46.05th percentile among solid tumors (Figure 3B). Microsatellite instability analysis confirmed a microsatellite-stable phenotype, and PD-L1 IHC (DAKO 22C3 assay) revealed both a tumor proportion score (TPS) and a combined positive score (CPS) of <1%. Collectively, these findings supported a diagnosis of metastatic pancreatic adenocarcinoma driven by an epigenetic alteration in *ARID1A* in conjunction with DNA amplifications at the 11q13.4 locus.

To evaluate the clinical impacts of *CCND1*/*FGF3*/*FGF4*/*FGF19* co-amplification, data from The Cancer Genome Atlas (TCGA), encompassing 20 cancer types, were analyzed to assess the overall survival (OS) in tumors harboring these alterations. co-amplification was most prevalent in head and neck squamous cell carcinoma, esophageal carcinoma, breast cancer, bladder cancer, and cholangiocarcinoma (Figure 3C). The top five frequent cancer types showed poor OS with the co-amplification (Figure 3D–H). In contrast, pancreatic cancer only ranked fifteenth, with only two cases exhibiting the co-amplification with OS 23 and 12 months, respectively, compared to a mean OS of 18.65 months in patients without co-amplification. These results suggested that *CCND1*/*FGF3*/*FGF4*/*FGF19* co-amplification may be associated with a poor prognosis.

The identified mutations in patients may offer critical guidance in selecting optimal therapeutic strategies. The patient harbored a germline *CHEK2* mutation, which is involved in DNA damage repair via the PARP1/2/3 pathway and may confer sensitivity to PARP inhibitors such as Talazoparib and Rucaparib, currently under clinical evaluation in breast, gynecologic, and other solid tumors. *ARID1A* mutation, associated with defective chromatin remodeling and aberrant activation of the mTOR pathway, suggested the potential responsiveness to mTOR inhibitors such as Everolimus, which is being investigated in cervical cancer for phase I, colorectal cancer, renal cell carcinoma, and non-small-cell lung cancer phase II. In addition, *CCND1* amplification indicates possible benefit from CDK4/6 inhibitors such as Abemaciclib or Palbociclib, both of which are approved in HR-positive and HER2-negative breast cancer. Furthermore, *FGF19* amplification is now being studied in phase I b/II trials for the experimental drug Fisogatinib (Figure 3I). Although these therapies are not yet approved for metastatic pancreatic adenocarcinoma, this integrated genomic profile highlighted multiple potential alterations and the value of comprehensive molecular profiling in guiding individualized therapy.

### 2.5. Clinical Diagnostic Pathway and Outcome

In the routine diagnostic pathway, conventional assessments—including PET/CT, contrast-enhanced CT, MRI, and laboratory tests such as CA 19-9—revealed multiple retroperitoneal soft tissue lesions and persistently elevated tumor markers, suggesting malignancy.

An ultrasound-guided needle biopsy from the peripancreatic mass was performed on 10 March 2023, and the histopathological results, available on 16 March 2023, revealed poorly differentiated adenocarcinoma. However, IHC analysis failed to identify the tissue of origin, resulting in diagnostic uncertainty. On 20 March 2023, an intraoperative frozen section again confirmed poorly differentiated adenocarcinoma. Nevertheless, the comprehensive IHC panel on the surgical resection specimen from the ligamentum teres hepatis and surrounding tissue was not finalized until 30 March 2023. The IHC profiles of both the needle biopsy and the surgical specimen were largely consistent. Tumor cells showed strong positivity for CK7, CK19, MUC-1, and DNA mismatch repair proteins (MLH1, PMS2, MSH2, MSH6), with partial positivity for CDX2 and Mesothelin, focal staining for Pax8, and Ki-67 (80%). Markers such as CK20, MUC-2, MUC-5AC, P40, and D2-40 were negative. In addition, the initial needle biopsy showed AE1/AE3 positivity and LCA positivity in lymphocytes, with negative staining for chromogranin A (CgA) and synaptophysin (Syn), excluding neuroendocrine differentiation. The surgical specimen additionally exhibited positivity for BerEP4 and negativity for SATB2 and GATA3 (Supplementary Figure S1). Despite multiple rounds of histopathology, no definitive origin could be determined. Collectively, the morphologic and IHC findings supported a diagnosis of cancer of unknown primary (CUP).

**Figure 4:**
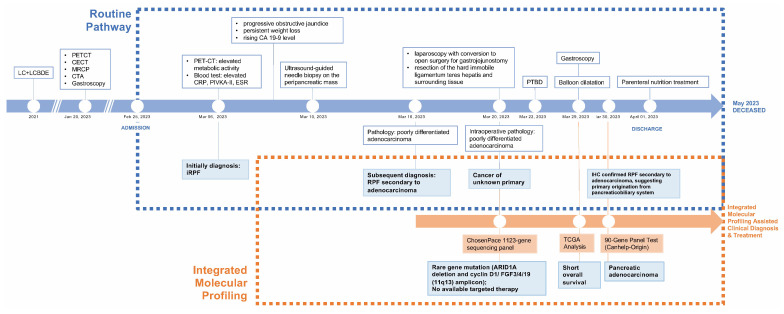
Clinical timeline and diagnostic pathway. Timeline highlighting key clinical events and the comparative impacts of routine versus multidisciplinary molecular profiling pathway.

To resolve this challenge, we initiated molecular profiling following the intraoperative identification of poorly differentiated adenocarcinoma from ligamentum teres hepatis tissue on 22 March 2023. Targeted next-generation sequencing using a 1123-gene panel identified a truncating mutation in *ARID1A* (NM_006015: c. 4336C>T (p. R1446*)) and co-amplification at the 11q13 locus (*CCND1*/*FGF3*/*FGF4*/*FGF19*). Following this, on 29 March 2023, we correlated the identified 11q13 co-amplified genes with overall survival using TCGA pancreatic cancer data, revealing a potential association with poorer prognosis. To complete these findings and reinforce the IHC-based suspicion of RPF secondary to pancreaticobiliary adenocarcinoma, we performed the Canhelp-Origin 90-gene expression classifier which yielded a similarity score of 45.5 for pancreatic tissue, further supporting a pancreatic origin. This molecular diagnostic triad—genomic mutation profiling, transcriptomic classification, and tissue-of-origin prediction—enabled a definitive diagnosis of metastatic pancreatic adenocarcinoma. This integrated approach not only clarified the tumor’s origin but also provided clinically relevant prognostic information, thereby informing precision management in this diagnostically challenging case (Figure 4).

Given the extent of advanced metastatic disease and extensive retroperitoneal fibrosis, curative resection was not feasible. The patient was provided with nutritional support, anti-infective therapy, and electrolyte balance maintenance. Hyponatremia and hypokalemia were corrected, and gastrojejunostomy (bypass) alleviated gastrointestinal discomfort. Owing to the patient’s poor tolerance, palliative chemotherapy was not instituted. Percutaneous transhepatic biliary drainage (PTBD) was then conducted to alleviate the aggravating obstructive jaundice. The patient later presented with dysphagia and vomiting. Upper gastrointestinal double-contrast radiography revealed lower esophageal obstruction and cardiac stenosis. Balloon dilation was performed to address these complications. The patient was discharged in a stable condition, maintaining basic total parenteral nutrition treatment, and eventually passed away two months post-discharge.

## 3. Discussion

This case illustrates the challenges of efficiently establishing a diagnostic pathway to minimize diagnosis delays in a patient with sRPF, complicated by congenital pancreatic hypoplasia and multiple hypometabolic peri-pancreatic and retroperitoneal masses. Based on the patient’s clinical presentations, laboratory findings, medication history, and imaging features, an initial hypothesis of heterotaxy polysplenia syndrome with benign iRPF was proposed, and steroid therapy was considered. However, the development of obstructive jaundice, worsening gastrointestinal symptoms, persistent weight loss, and elevating CA 19-9 prompted an ultrasound-guided needle biopsy of the peripancreatic mass, which revealed poorly differentiated adenocarcinoma, with a suspected primary origin in pancreaticobiliary system. Nevertheless, conventional histopathology and immunohistochemistry alone failed to definitively determine the primary tumor site, highlighting diagnostic limitations and emphasizing the critical role of molecular diagnostics.

### 3.1. Limitations of Routine Clinical Pathway in Differential Diagnosis

Differential diagnosis of RPF remains a major clinical challenge, particularly in cases with atypical presentations such as maRPF [16]. While iRPF is commonly associated with autoimmune or IgG4-related disease, maRPF may present with nonspecific clinical features that mimic benign conditions, resulting in misdiagnosis and delays in initiating appropriate treatment [17]. This case exemplifies a rare scenario in which pancreaticobiliary malignancy initially manifested as iRPF, further complicating timely diagnosis.

Specifically, although the patient exhibited elevated CRP, ESR, and positive anti-nuclear antibody levels, the degree was insufficient to definitively guide the diagnostic process. Despite atypical clinical findings, including laboratory assessments, the absence of definitive radiological or histopathological evidence of malignancy at the initial assessment likely led to a delay in identifying a malignancy.

Although 18F-FDG PET/CT is useful in evaluating both metabolic and inflammatory activity, it lacks sufficient specificity to confirm malignancy [7,18]. This highlights a critical diagnostic challenge: when clinical, laboratory, and imaging findings deviate from the classical presentation of iRPF, clinicians should maintain a high index of suspicion for secondary causes and prioritize early tissue biopsy to rule out malignancy, particularly in diagnostically ambiguous cases.

As suggested, tissue biopsy from the site showing the greatest lesion thickness and the highest hypermetabolism on PET is generally recommended for definitive diagnosis under the following conditions: (i) when the mass is located atypically, (ii) when clinical and laboratory findings suggest malignancy or infection, and (iii) when there is a bulky retroperitoneal mass with infiltration into muscles, bone, or other structures, or in cases of confluent lymphadenopathy [8].

### 3.2. Considerations Regarding Cancer of Unknown Primary (CUP)

Despite obtaining tissue biopsy from the retroperitoneal mass, histopathological analysis only confirmed poorly differentiated adenocarcinoma without identifying the primary tumor origin. These factors led to the diagnostic ambiguity, ultimately classifying the case as CUP.

For CUP, traditional pathology and imaging techniques have a diagnostic accuracy of only 20% to 30% [18]. Histopathological diagnosis of CUP is often delayed significantly, due to the need for sequential tissue sampling, tumor heterogeneity, differences in tissue antigenicity, and the subjective interpretation of pathological features. Although ^18^F-FDG PET/CT has improved the detection rate of CUP, it has limited sensitivity in detecting small or low-activity lesions [18]. These limitations highlight the need for molecular approaches to enhance diagnostic precision. Additional clinically validated tests have therefore been proposed to facilitate final diagnosis [19].

One such tool is the 90-gene panel test known as Canhelp-Origin and developed by Fudan University. It is approved by the National Medical Products Administration (NMPA) for identifying rare tumor types [20]. The predefined 90-gene classifier analyzes gene expression patterns to generate probability-based similarity scores for 21 primary tumor types, with scores summing to 100 for each sample [21]. The tumor type with the highest similarity score is considered the predicted origin, achieving an overall accuracy of 90% for both primary and metastatic tumors [22]. In the present case, the application of this tool significantly reduced diagnostic ambiguity, supporting the final diagnosis of metastatic pancreatic adenocarcinoma with a high similarity score.

### 3.3. Considerations Regarding Prognosis and Targeted Therapy

Although the tumor origin was successfully traced, the incomplete mutational profile has limited our ability to develop precise, targeted therapeutic strategies. The ChosenPace 1123-gene sequencing panel identified *CCND1/FGF3/FGF4/FGF19* co-amplification at the 11q13 locus. This combination of genetic alterations has been commonly observed in cancers such as head and neck squamous cell carcinoma, esophageal carcinoma, breast cancer, bladder cancer, and liver hepatocellular carcinomas, aligning with findings from the TCGA database [23,24,25,26,27,28]. In pancreatic cancer, however, such co-amplifications are relatively rare. While *ARID1A* mutations occur in approximately 4–8% of pancreatic adenocarcinoma, they are associated with chromatin remodeling defects and may contribute to tumor aggressiveness when co-occurring with oncogenic amplifications. The co-occurrence of *ARID1A* deletion and *CCND1/FGF3/FGF4/FGF19* co-amplification in this case suggests a particularly aggressive molecular phenotype, with a potential synergistic effect contributing to both tumor aggressiveness and poor prognosis. Further research is needed to explore targeted therapeutic strategies for tumors harboring *CCND1/FGF3/FGF4/FGF19* co-amplifications and *ARID1A* alterations.

### 3.4. Limitations of the Current Case Report and Future Direction

Several limitations in clinical practice on such complicated cases should be acknowledged. First, routine molecular profiling techniques and tumor origin-tracing strategies are not yet widely implemented in clinical practice. Additionally, larger cases cohorts are needed to develop a robust prognostic prediction model. As an emerging technology, artificial intelligence (AI)-assisted pathology detection shows considerable potential to enhance diagnostic efficiency [18]. A recent systematic review and meta-analysis reported that AI applied to digital pathology images achieved a mean sensitivity of 96.3% and a mean specificity of 93.3% across various disease types, underscoring its promise in improving diagnostic performance [29].

## 4. Methods

### 4.1. Study Approval

A three-generation pedigree with eight family members were recruited from the Beijing Tsinghua Changgung Hospital of Tsinghua University. This study was approved by the Ethical Review Board of Beijing Tsinghua Changgung Hospital, Tsinghua University. Informed consent was obtained from the patient prior to publication of this report.

### 4.2. Sample Processing and DNA Extraction

Tissue samples were obtained from formalin-fixed, paraffin-embedded (FFPE) blocks and sectioned to a thickness of 10 μm. Paraffin was removed by incubating the sections in xylene for 10 min, followed by rehydration through a graded ethanol series. DNA was then extracted using the QIAamp DNA FFPE Tissue Kit (QIAGEN, Hilden, Germany). Briefly, the extraction protocol involved overnight lysis in ATL buffer containing proteinase K at 56 °C, binding of the lysate to a QIAamp Mini column after the addition of AL buffer and ethanol, washing with AW1 and AW2 buffers, and elution in AE buffer. DNA concentration and purity were assessed using a NanoDrop spectrophotometer (Thermo Fisher Scientific, Wilmington, DE, USA).

### 4.3. Library Preparation and Targeted Sequencing

For targeted sequencing, 500 ng of genomic DNA from each sample was fragmented to 200–250 base pairs using a Covaris M220 sonicator (Covaris, Woburn, MA, USA). The fragmented DNA underwent end repair, A-tailing, and ligation to indexed adapters with the KAPA Hyper Prep Kit (Roche Diagnostic, Basel, Switzerland). The adapter-ligated libraries were purified with AMPure XP beads (Beckman Coulter, Indianapolis, IN, USA) and quantified using a Qubit fluorometer (Thermo Fisher Scientific, Wilmington, DE, USA). Target enrichment was performed using the ChosenPace 1123-gene panel. Libraries were hybridized to the panel probes for 16 h at 65 °C, washed with the provided buffers, and amplified for 12 PCR cycles using KAPA HiFi HotStart ReadyMix (Roche Diagnostic, Basel, Switzerland). Sequencing was conducted on an MGISEQ2000 platform (MGI Tech Co., Ltd., Shenzhen, China) with paired-end 100 bp reads. Raw sequencing data were processed through the ChosenPace bioinformatics pipeline, achieving a raw depth exceeding 2000× and an effective depth above 500×.

### 4.4. Next-Generation Sequencing Data Processing and Variant Calling

Raw FASTQ files were processed using Fastp (v0.23.0) for quality control, yielding clean reads that were aligned to the UCSC hg19 human reference genome with BWA (v0.7.11). Somatic mutations—including small insertions, deletions, and single nucleotide polymorphisms (SNPs)—were detected using GATK IndelRealigner (v4.2) and VarScan with default settings. Variants were annotated using ANNOVAR (v2020-06-08) against databases such as dbSNP (build 147), ClinVar, COSMIC (v70), MutationTaster, CADD, and the 1000 Genomes Project. Candidate mutations were filtered by excluding intronic variants, variants with a population frequency > 1% in the 1000 Genomes Project or ExAC_all, common SNPs in dbSNP, synonymous variants, and those supported by fewer than 8 reads. Tumor mutational burden (TMB) was calculated as the number of non-synonymous somatic mutations in the coding regions captured by the panel.

### 4.5. 90-Gene Panel Test (Canhelp-Origin)

Between five and fifteen unstained 5 μm FFPE sections were prepared for analysis. H&E-stained slides were used to evaluate tumor cell content, delineate tumor regions, and guide manual macrodissection for enrichment. Total RNA was extracted using the FFPE Total RNA Isolation Kit (Canhelp Genomics Co., Ltd., Hangzhou, China) following established protocols, and RNA concentration and purity were measured by spectrophotometry. The 90-gene expression assay (Canhelp Genomics Co., Ltd.) was performed according to standard protocols, involving reverse transcription of total RNA and real-time PCR for comprehensive tumor-specific gene expression profiling. Assay results were accepted only if the internal control cycle threshold (Ct) values were below 38 and the no-template control Ct values were above 38. For each case, the prespecified 90-gene classifier was applied to analyze gene expression patterns and generate probability-based similarity scores for 21 primary tumor types, with scores ranging from 0 to 100 and summing to 100 per sample. The tumor type with the highest similarity score was designated as the predicted origin.

### 4.6. CCND1/FGF3/FGF4/FGF19 Co-Amplification and Survival Analysis in TCGA

Individuals harboring co-amplifications of *CCND1/FGF3/FGF4/FGF19* were identified from the TCGA PanCancer Atlas via the cBioPortal platform (https://www.cbioportal.org/). The top five cancer types with the highest frequency of these co-amplifications were selected for further analysis. Overall survival (OS) was compared between co-amplification carriers and non-carriers using the Kaplan–Meier method. Differences in survival distributions were assessed using the log-rank test. A *p*-value < 0.05 was considered statistically significant.

## 5. Conclusions

This case illustrates the diagnostic challenges of maRPF with atypical fibrotic manifestations. Routine clinical evaluations, including serological markers, imaging studies, and histopathological analysis, were insufficient to determine the tumor’s origin, delaying both diagnosis and treatment. Our findings demonstrate that a multidisciplinary approach incorporating molecular profiling is essential for refining the diagnostic pathway. This strategy not only enhanced differential diagnosis but also provided a robust molecular foundation for the development of personalized therapeutic strategies. Consequently, such an integrated model should be considered not only for rare pathological presentations but also more broadly in oncologic cases where conventional diagnostics fall short.

## Data Availability

Data are not publicly available in order to protect patient privacy. The ethics committee and informed consent does not allow for these data to be deposited into a secure access-controlled repository. Qualified researchers can apply for access to the data by contacting the corresponding authors. The data will be made available on reasonable request.

## Supplementary Materials

The following supporting information can be downloaded at https://www.mdpi.com/article/10.3390/diagnostics15161998/s1: Figure S1: Immunohistochemical (IHC) staining results of the retroperitoneal mass resected from the ligamentum teres hepatis. Representative IHC images from the surgical specimen are shown, including positive markers (left column), corresponding negative controls (middle column), and negative markers (right column). The tumor cells demonstrated strong positivity for CK7, CK19, MUC1, MLH1, PMS2, MSH2, MSH6, BerEP4, and MOC-31; partial or focal positivity for CDX2, CEA, Mesothelin, CR, Pax8, and WT1; and a proliferation index of approximately 80% as indicated by Ki-67. Markers for other site-specific differentiation—including CK20, MUC2, MUC6, MUC-5AC, P40, HER2, EGFR, D2-40, SATB2, and GATA3—were negative. Negative controls showed no nonspecific staining, confirming the specificity of each antibody used.
