# Supplementary material for: ARID1A-Deficient and 11q13-Amplified Metastatic Pancreatic Cancer Initially Presenting as Retroperitoneal Fibrosis in a Patient with Familial CHEK2 Variant
Source: Diagnostics (Basel). 2025 Aug 9;15(16):1998. doi: 10.3390/diagnostics15161998 (PMC12385468; doi:10.3390/diagnostics15161998)
Supplement: Supplementary file 1 [file diagnostics-15-01998-s001.zip › diagnostics-3649537-supplementary.pdf]

Supplementary Information

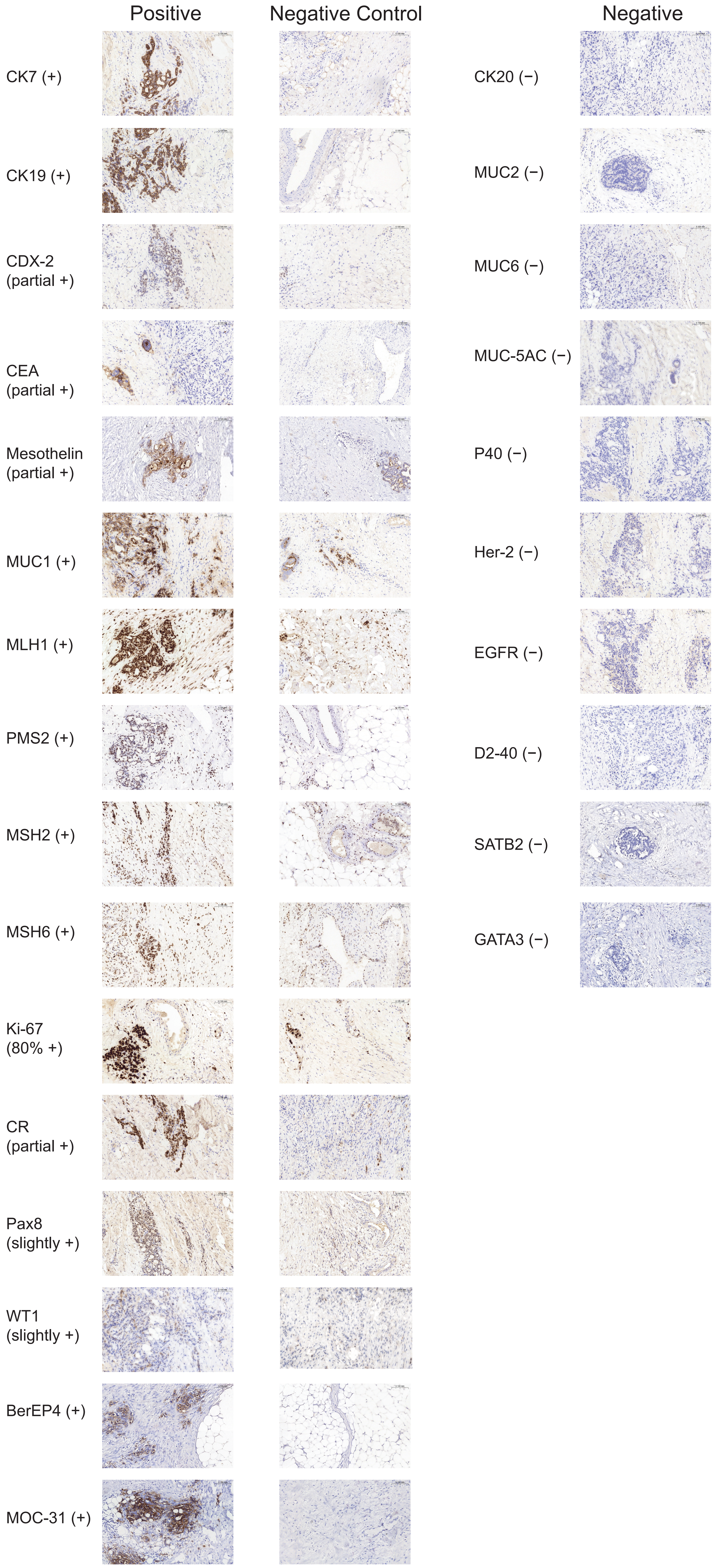

Figure S1: Immunohistochemical (IHC) staining results of the retroperitoneal mass resected from the ligamentum teres hepatis. Representative IHC images from the surgical specimen are shown, including positive markers (left column), corresponding negative controls (middle column), and negative markers (right column). The tumor cells demonstrated strong positivity for CK7, CK19, MUC1, MLH1, PMS2, MSH2, MSH6, BerEP4, and MOC-31; partial or focal positivity for CDX2, CEA, Mesothelin, CR, Pax8, and WT1; and a proliferation index of approximately 80% as indicated by Ki-67. Markers for other site-specific differentiation—including CK20, MUC2, MUC6, MUC-5AC, P40, HER2, EGFR, D2-40, SATB2, and GATA3—were negative. Negative controls showed no nonspecific staining, confirming the specificity of each antibody used.
